# Supplementary material for: Comparative micromechanics of bushcricket ears with and without a specialized auditory fovea region in the crista acustica
Source: Proc Biol Sci. 2020 Jun 24;287(1929):20200909. doi: 10.1098/rspb.2020.0909 (PMC7329045; doi:10.1098/rspb.2020.0909)
Supplement: Supplement Figures for Broadness and AUC [file rspb20200909supp4.docx]

**
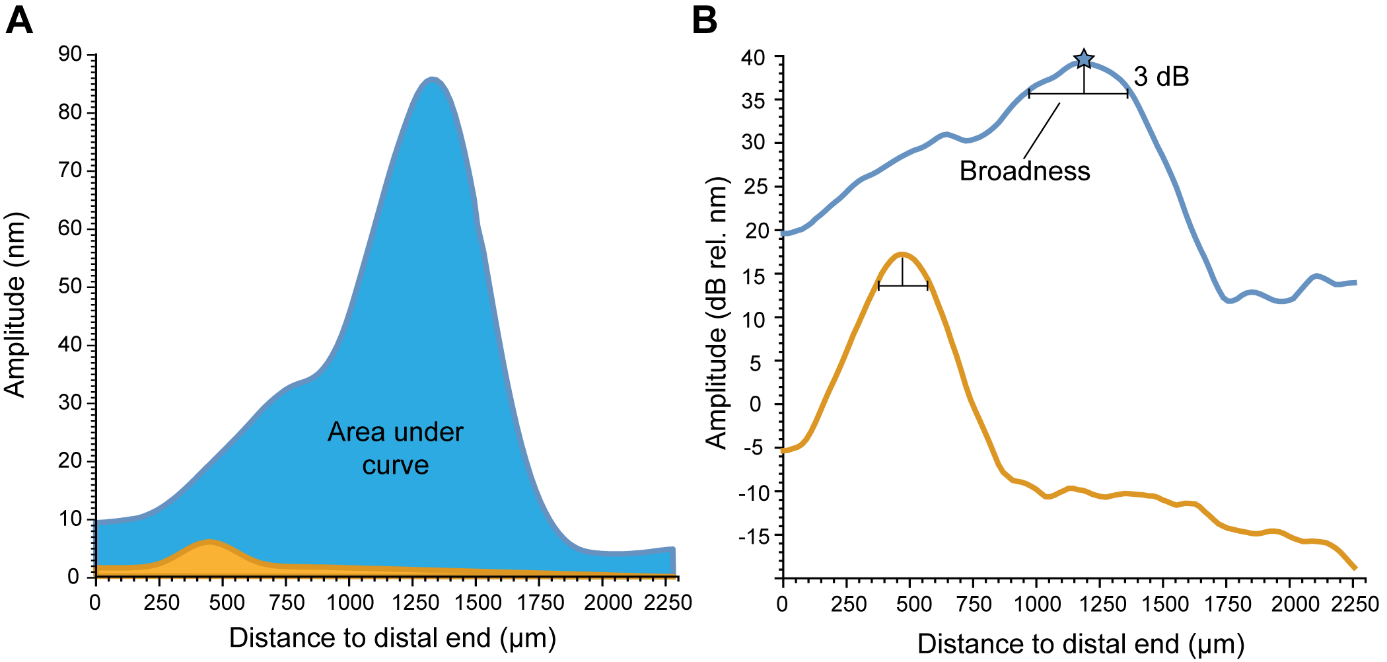
Supplement Figure 1:** **Calculation of curve broadness and area under curve.**

A) The broadness of the response is calculated 3 dB below the position of highest amplitude for each stimulation frequency. B) The area under curve is calculated for each frequency response and the results are shown in Figure 5.


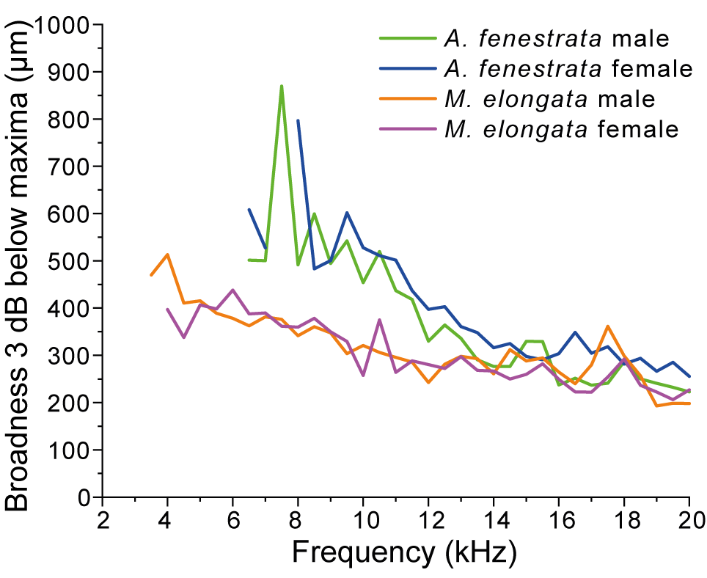


**Supplement Figure 2: Broadness of mechanical response curves.**

Additional to the height of the mechanical response, the broadness of the amplitude was calculated to distinguish between an overall amplitude amplification in the foveal region or just increased height. Broadness of the amplitude response curves shown in Figures 3 and 4 calculated 3 dB below the maximum for each tested frequency, respectively. Low broadness values mean a stimulation of only a small *crista acustica* region and therefore just a few reacting sensory units for a specific frequency. Please note that for low frequency stimulation curve broadness couldn’t always be calculated.
